# Prevalence of celiac disease in adult type 1 patients with diabetes

**DOI:** 10.12669/pjms.314.7206

**Published:** 2015

**Authors:** Burcu Dogan, Can Oner, Oya Uygur Bayramicli, Elif Yorulmaz, Guneş Feyizoglu, Aytekin Oguz

**Affiliations:** 1 Burcu Dogan, Department of Family Medicine, Istanbul Medeniyet University, Goztepe Training and Research Hospital, Istanbul Turkey; 2 Can Oner, Istanbul Bilim University, School of Medicine, Department of Family Medicine, Istanbul Turkey; 3 Oya Uygur Bayramicli, Private office, Istanbul Turkey; 4 Elif Yorulmaz, Istanbul Bagcılar Traning and Research Hospital, Department of Gastroenterology, Istanbul Turkey; 5 Guneş Feyizoglu, Department of Internal Medicine, Istanbul Medeniyet University, Goztepe Training and Research Hospital, Istanbul Turkey; 6 Aytekin Oguz, Department of Internal Medicine, Scholl of Medicine, Istanbul Medeniyet University, Istanbul Turkey

**Keywords:** Type 1 diabetes, Celiac disease, Prevalence

## Abstract

**Objectives:**

Celiac disease, an autoimmune disease, is related to immune mediated intolerance to gluten. Some studies suggest that Celiac Disease was 20 times more frequent in type 1 patients with diabetes. The objective of our study was to evaluate the prevalence of celiac disease in hospital based type 1 diabetic adults.

**Methods:**

Our study was carried out retrospectively in Medeniyet University Goztepe Training and Educational Hospital in Istanbul between 2012–2013. The cohort comprised 482 type 1 patients with diabetes attending the diabetes outpatient clinic. The data were analyzed by SPSS 10.5 package program. Student’s t tests is used for comparative analyses. A p-value less than 0.05 was considered statistically significant.

**Results:**

The cohort included 482 type 1 patients with diabetes. Fifty seven of them were not evaluated for Endomysium antibody positivity. Fifteen of the remaining 425 patients were positive for anti endomysial antibody (3.5%). The prevalence of biopsy proven celiac disease was 2.3% (10/425). There was no significant difference between Endomysial antibody positive and negative groups in regard of age, sex, or duration of the disease.

**Conclusion:**

This study confirms that the celiac disease is common in type 1 diabetic patients. Since a small proportion of celiac patients are symptomatic this disorder should be screened in all adult type 1 patients with diabetes by antiendomysium antibody.

## INTRODUCTION

Celiac disease (CD), an autoimmune disease, was related with immune mediated intolerance to gluten. This intolerance leads to immune mediated inflammatory damage to intestinal epithelium. The typical form of the disease is seen in only 30-40% of the patients.^1^ Nowadays studies using antibodies with biopsy verification, report rates 1:120 to 1:300 in most countries in normal population.^2^-^4^ In Turkey it was estimated that the prevalence of CD was 1:87 (1.2%).^5^

First in 1969 the association between Celiac Disease and Type 1 DM was identified.^6^ After that many studies also reveals the relation between CD and Type 1 DM. Recent studies reveals that 1-8% of the type 1 diabetics have CD.^7^-^9^ Also some studies suggest that CD was 20 times more frequent in type 1 diabetics.^10^-^11^ A study conducted in Turkey found CD prevalence in adult type 1 diabetes as 6%.^12^ It was assumed that half of the patients remain asymptomatic.^13^ Clinically silent CD patients are diagnosed most of the times serological screening or during endoscopy and biopsy for another reason. It was estimated that the disease is more frequent and can sometimes present with atypical symptoms like iron deficiency anemia, infertility, malignancy or neurological disorders.^14^

Many studies have been performed to evaluate the efficacy of screening CD in type 1 diabetes. The physician should be suspicious for diagnosis of CD. Suspected patients can be screened with anti endomysium antibodyies. Near 5–10% of patients with type 1 diabetes were positive for EMA antibodies, and a significant proportion have also abnormalities on biopsy of the intestine.^15^ But important part of type 1 diabetic patients were negative in first screen for CD and become positive later.^8^ So it can be suggested that single screening is not effective for CD. On the other hand antibody positivity do not increase risk of abnormalities on biopsies. Both normal and diabetic patients with antibody positivity the rates of biopsy abnormalities were estimated as 75%.^16^ Today screening of all the type 1 diabetics for antibody positivity at diagnosis and presence of symptoms is recommended. Moreover antibody positive subjects should be examined by biopsy to confirm diagnosis.^15^

The objective of our study was to evaluate the prevalence of celiac disease in type 1 diabetic adults in a hospital based cohort.

## METHODS

Our study was carried out retrospectively in Medeniyet University Goztepe Training and Educational Hospital in Istanbul between 2012-2013. The cohort composed of 482 type 1 diabetic patients (264 males and 218 females) attending the diabetes outpatient clinic. Inclusion criteria were as follows; 1) Age between 15- 80 years, 2) onset of diabetes before 30 year of age, 3) history of diabetic ketosis and 4) unbroken record of insulin treatment from the initial diagnosis. The records of patients was evaluated.

Antiendomysium antibodies (Anti EMA) were determined by indirect immunoflorescense antibody testing. The defined cut-off point for positivity was 5 U/ml. Patients positive for antiendomysial antibodies were informed about the results and referred to the department of gastroenterology for upper gastrointestinal endoscopy with duodenal biopsy. The study group had been scoped by the same endoscopist with a Fujinon CV-160 videogastroscope in a standard fashion and 6 biopsies were taken from the second portion of the duodenum and were sent for histopathological evaluation. For the pathological evaluation of endoscopic biopsies standard criteria defined by Marsch were used.^17^ The data were analyzed by SPSS 10.5 package program. The t test used for comparative analyses. A p value less than 0.05 was considered statistically significant.

## RESULTS

The cohort included 482 type 1 diabetic patients. Fifty seven of them were not evaluated for anti EMA positivity.15 of the remaining 425 patients were positive for anti endomysial antibody (3.5%). One of the patients was not anti EMA positivite but she was symptomatic for celiac disease. Fourteen patients underwent upper gastrointestinal endoscopy and distal doudenal biopsies were taken. Morphologic changes were consistent with celiac disease in 10 of them. Doudenal biopsy samples of these patients revealed grade 3a in 6 patients and 3b in 4 patients according to modified Marsh classification (Table-I). The prevalence of biopsy proven celiac disease was 2.3% (10/425). The clinical and paraclinical characteristics of the patients with CD is summarized in Table-II. Six of the patients were male and remaining 4 was female. Mean age of patients was 36.1±10.3 year. One of the patients complained about abdominal bloating, nausea and diarrhea. Remaining patients were asymptomatic. One patient had iron deficiency anemia.

**Table-I:** Patients distribution according to Marsh criteria.

Marsh Grade	Patients (n, %)
0	0
1	0
2	0
3a	6
3b	4
3c	0

**Table-II:** Clinical characteristics of 10 patients with CD.

	Patients									
	1	2	3	4	5	6	7	8	9	10
Age (year)	37	47	29	37	46	26	28	39	52	20
Sex	M	M	M	F	F	M	M	F	M	M
BMI (kg/m^2^)	22,7	26,3	23,2	20,9	22,4	25,9	24,1	26,0	23,7	22,9
Hg (mg/dl)	14,7	13,8	14,4	9,5	12,7	15,1	15,7	12,7	12,8	14,9
HbA1c (%)	9,2	8,3	6,0	8,4	6,3	11,3	14,2	7,3	8,7	6,9
Diabet duration (year)	28	38	18	30	14	2	25	24	24	1
Marsh Classification	3b	3a	3a	3b	3a	3a	3a	3a	3a	3b

There were no significant difference between two groups as regards age, sex, or duration of disease. There clinical features of EMA positive and EMA negative group are summarized in Table-III.

**Table-III:** Characteristics of the cohort.

	EMA negative group (n= 410)	EMA positive group (n=15)	P value
Female	45,8 % (n=188)	40 % (n=6)	NS
Male	54,2 % (n=222)	60% (n=9)	NS
Age (year)	36,17±11,03	39,20±8,66	NS
Age (Female)	35,99±11,25	38,83±4,07	NS
Age (Male)	36,32±10,86	39,44±10,99	NS
Diabetes duration (year)	23,80±11,86	25,40±10,04	NS
HbA1c (%)	8,70±2,12	8,77±2,44	NS

## DISCUSSION

Prevalence of CD was about 1% in Turkey.^18^ In this cross sectional study we determined the overall prevalence of celiac disease in type 1 diabetic adults was nearly 2.3% (10/425). Except one patient all were asymptomatic and did not manifest any clear symptoms of CD. The prevalence of disease in adult type 1 diabetes was similar in European countries with a range of 1-7.8%.^9^ In middle east countries the prevalence of CD ranges between 3.5%-15%.^19^ A study conducted in Turkey found CD prevalence in adult type 1 diabetes as 6%.^12^

Like other studies nearly all participants of our study were not suspected to have celiac disease because of the lack of symptoms like diarrhea, weight loss or abdominal distension.^13^,^19^-^20^ It is well known that in patients with type 1 diabetes the symptoms of celiac disease are absent.^21^ So even a careful patient history can underestimate the frequency of celiac disease in patients with type 1 diabetes.

The diagnosis of CD requires to be suspicious. All the patients can be screened for CD by autoimmunity with anti endomysial antibody (EMA). EMA are autoantiboides against antigens in the collageneous matrix. The sensitivity is about 90% and specificity approaches to 100%.^22^ But definitive diagnosis of CD is obtained by small intestine biopsies. Up to 5-10% of type 1 diabetics have positive EMA antibodies and nearly 75% of them have abnormalities on small intestinal biopsy.^15^ We detected EMA positivity as 3.5% in type 1 diabetic patients.

Because the prevalence of CD is higher in type 1 diabetic patients the efficacy of screening of CD in this population has remained under discussion. The current recommendation for screening CD in type 1 diabetics are obtaining auto antibodies at the diagnosis of diabetes. Subjects with positive antibodies should be screened by small intestinal biopsies to confirm diagnosis.^15^ Most of the patients are diagnosed as suffering from diabetes and then CD as such screening should be continued for up to six years.^22^,^23^ However, our data suggest to continue screening for more than 6 years, because most of the patients were diagnosed with CD after 10-15 years of being diagnosed to be of type 1 diabetes and delay of CD diagnose was frequently present.

## CONCLUSION

This study confirms that the celiac disease is common in type 1 patients with diabetes. The prevalence of celiac disease among low risk populations was 1-1.3%.^13^,^14^ Since a small proportion of celiac patients are symptomatic this disorder should be screened in all adult type 1 diabetics by antiendomysium antibody.
